# An RCT into the effects of neurofeedback on neurocognitive functioning compared to stimulant medication and physical activity in children with ADHD

**DOI:** 10.1007/s00787-016-0902-x

**Published:** 2016-09-24

**Authors:** Katleen Geladé, Marleen Bink, Tieme W.P. Janssen, Rosa van Mourik, Athanasios Maras, Jaap Oosterlaan

**Affiliations:** 1 Yulius Academy, Yulius Mental Health Organization, Dennenhout 1, 2994 GC Barendrecht, The Netherlands; 2 Department of Clinical Neuropsychology, VU University Amsterdam, Van der Boechorstraat 1, 1081 BT Amsterdam, The Netherlands; 3 Royal Dutch Kentalis, Utrecht, Pallas Athenedreef 10, 3561PE Utrecht, The Netherlands

**Keywords:** ADHD, Neurocognition, Medication, Neurofeedback, Physical activity

## Abstract

Neurofeedback (NFB) is a potential alternative treatment for children with ADHD that aims to optimize brain activity. Whereas most studies into NFB have investigated behavioral effects, less attention has been paid to the effects on neurocognitive functioning. The present randomized controlled trial (RCT) compared neurocognitive effects of NFB to (1) optimally titrated methylphenidate (MPH) and (2) a semi-active control intervention, physical activity (PA), to control for non-specific effects. Using a multicentre three-way parallel group RCT design, children with ADHD, aged 7–13, were randomly allocated to NFB (*n* = 39), MPH (*n* = 36) or PA (*n* = 37) over a period of 10–12 weeks. NFB comprised theta/beta training at CZ. The PA intervention was matched in frequency and duration to NFB. MPH was titrated using a double-blind placebo controlled procedure to determine the optimal dose. Neurocognitive functioning was assessed using parameters derived from the auditory oddball-, stop-signal- and visual spatial working memory task. Data collection took place between September 2010 and March 2014. Intention-to-treat analyses showed improved attention for MPH compared to NFB and PA, as reflected by decreased response speed during the oddball task [*η*_p_^2^ = 0.21, *p* < 0.001], as well as improved inhibition, impulsivity and attention, as reflected by faster stop signal reaction times, lower commission and omission error rates during the stop-signal task (range *η*_p_^2^ = 0.09–0.18, *p* values <0.008). Working memory improved over time, irrespective of received treatment (*η*_p_^2^ = 0.17, *p* < 0.001). Overall, stimulant medication showed superior effects over NFB to improve neurocognitive functioning. Hence, the findings do not support theta/beta training applied as a stand-alone treatment in children with ADHD.

**Electronic supplementary material:**

The online version of this article (doi:10.1007/s00787-016-0902-x) contains supplementary material, which is available to authorized users.

## Introduction

Attention deficit hyperactivity disorder (ADHD) is a highly prevalent neurodevelopmental disorder [1] characterized by age-inappropriate symptoms of inattention, hyperactivity and impulsivity [2]. Impaired neurocognitive functioning is considered a core dysfunction of the disorder [3, 4] and is reflected in deficiencies in a variety of neurocognitive functions including attention, inhibition, and working memory [5–8]. Stimulant medication is a commonly used and effective treatment in reducing behavioral symptoms [9] and has also been found to improve neurocognitive functioning [10] in children with ADHD. However, the use of stimulant medication has several adverse side effects such as sleep problems, decreased appetite, and headaches [11]. Moreover, there is limited evidence of long-term efficacy of stimulant medication [12]. Neurofeedback has been proposed to be a potentially effective non-pharmacological treatment for ADHD with effects comparable to stimulant medication [13, 14].

Neurofeedback aims to optimize brain activity, by providing the patient with visual and/or auditory feedback of electroencephalogram (EEG) activity, which is suggested to result in enhanced neurocognitive functioning that in turn translates into improved behavioral functioning [15]. Children with ADHD have been found to show increased theta (4–8 Hz) and decreased beta (13–20 Hz) activity in EEG measures of brain activity [16]. Increased theta and decreased beta activity have been related to poor vigilance and reduced attention, respectively [17]. A protocol that is often used in ADHD treatment, aims at decreasing theta activity and increasing beta activity [14, 18].

Results of randomized controlled trials (RCTs) on the effects of neurofeedback are mixed for neurocognitive outcome measures [19–27]. Overall, double-blinded RCTs revealed no additional effect of neurofeedback over sham-neurofeedback on neurocognitive measures [19, 23, 28]. One single-blinded RCT [20] showed superiority of neurofeedback over electromyography (EMG)-biofeedback on attention measures. Bink et al. [21] compared treatment as usual combined with neurofeedback to treatment as usual, and found no additional value of neurofeedback on measures of attention and working memory. In contrast, the study by Steiner et al. [29] found improved executive functioning in children with ADHD who received additional neurofeedback treatment compared to those who received only treatment as usual. A recent meta-analysis shows that overall neurofeedback does not induce significantly improved neurocognitive functioning compared to (active) control conditions [30]. However, studies comparing neurofeedback to stimulant medication on neurocognitive measures are scarce. There is only one RCT [31] that compared effects of neurofeedback with stimulant medication as stand-alone treatments on neurocognitive functioning, as assessed using measures of attention and executive functioning. In that study, the 16 children who received stimulant medication, showed larger improvement in neurocognitive functioning than the 16 children who received neurofeedback. However, the study was hampered by small sample size, a broad age range of participants (7 through 16 years), wide distribution of the 30 neurofeedback sessions over an intervention period of 6–9 months, and lack of transfer strategies into daily life. In the current study, we addressed these shortcomings, comparing neurofeedback to stimulant medication (short-acting methylphenidate), as stand-alone treatments on neurocognitive functioning. Furthermore, to control for non-specific treatment effects of neurofeedback, a physical activity training was applied as semi-active control condition. Accordingly, the physical activity training was matched to the neurofeedback training in terms of frequency and duration of the training sessions. By matching the training intensity of the neurofeedback and physical activity training, we aimed to control for non-specific treatment effects, such as parental engagement and personal attention. Using a multicenter three-way parallel RCT design, the aim of the present study was to compare the three treatments: neurofeedback (NFB), stimulant medication (MPH) and the semi-active control condition consisting of physical activity (PA), in terms of their effects on neurocognitive functioning, as assessed using measures of attention, inhibition, and visual spatial working memory.

## Methods

### Participants

Eligible participants were Dutch speaking children, 7–13 years of age, with a primary clinical DSM-IV-TR diagnosis of ADHD [2]. Children with ADHD were recruited from fifteen child mental health outpatient care facilities in the West of the Netherlands. Before entering the study, parent- and teacher ratings on the Disruptive Behavior Disorders Rating Scale (DBDRS) [32] confirmed their diagnosis; at least one of the scores on the Inattention or Hyperactivity/Impulsivity scales had to be above the 90th percentile for one of the informants, and above the 70th percentile for the other informant (signifying pervasiveness of symptoms). At study entry, all children were free of stimulant use for at least 1 month. Exclusion criteria were neurological disorders and IQ below 80 as measured by a four subtest version of the Wechsler Intelligence Scale of Children-III (WISC-III) including the subtests Vocabulary, Arithmetic, Block Design, and Picture Arrangement [33]. No restrictions were set on other comorbidities. Comorbid disorders were diagnosed according to DSM-IV-TR and retrieved from the medical records. Comorbid disorders included learning disorders (NFB; *n* = 5, MPH; *n* = 2, PA; *n* = 1), autism spectrum disorders, (NFB; *n* = 3, MPH; *n* = 2, PA; *n* = 3), anxiety disorders (NFB; *n* = 2, MPH; *n* = 0, PA; *n* = 2), and mood disorder (NFB; *n* = 1, MPH; *n* = 0, PA; *n* = 0). Chi-square test revealed no significant difference in the distribution of comorbid disorders over groups, *χ* ^2^ (8, *N* = 112) = 12.88, *p* = 0.12.

Initially, 112 children with ADHD were randomized over the three interventions (NFB; *n* = 39; MPH; *n* = 36; PA; *n* = 37), with 103 children completing their intervention (NFB; *n* = 38; MPH; *n* = 31; PA; *n* = 34). Drop-out reasons included motivational and/or practical reasons (NFB; *n* = 1, MPH; *n* = 3, PA; *n* = 3) and medical contraindications (MPH; *n* = 2). A participant flow diagram is presented elsewhere [34].

### Trial design

A multicentre three-way parallel group study with balanced randomization was conducted. A randomization table was created using a computerized random number generator [35]. Stocks of nine unmarked sealed envelopes were presented to parents at intake. Parents randomly picked an envelope revealing intervention allocation. Subsequently, children, parents, and teachers were aware of the allocated group. Data collection took place between September 2010 and March 2014.

The current study aimed to enroll 186 participants. In total, 135 children with ADHD were assessed for eligibility and eventually 112 participants were randomized over the three interventions. To detect a medium effect size (*f* = 0.25) using three groups in a repeated measures (RM) analysis of variance (ANOVA) with an alpha 0.05 and a power of 95 %, a total sample size of 66 (i.e. 22 per group) was required [36]. Post-hoc comparisons of two groups required a total sample size of 54 (i.e. 27 per group) to detect a medium effect size (*f* = 0.25) in a RM ANOVA with an alpha 0.05 and a power of 95 %. In the current study, the smallest group size was 29. Consequently, all groups had enough participants to detect a medium effect size. Because in total 112 participants were randomized over the three groups instead of 186 participants, the current study did not achieve the statistical power needed to detect smaller effect sizes than medium (*f* < 0.25) between groups. This report complies with the CONSORT 2010 guidelines (Supplement Appendix 1) for reporting parallel group randomized trials [37]. The trial was registered on clinicaltrials.gov (Ref. No. NCT01363544).

### Interventions

NFB and PA treatment consisted of three individual training sessions a week, with each session lasting 45 min including 20 min of effective training, over a period of 10–12 weeks. All interventions, as described below, took place after the pre-intervention (t0) assessment.

*Neurofeedback (NFB).* Theta/beta training was applied with the aim to inhibit theta (4–8 Hz) and reinforce beta (13–20 Hz) activity at Cz. Theta/beta index was represented to the participant by simple graphics on a screen. Successful reduction of the theta/beta index as averaged over one trial relative to session baseline, was rewarded with the appearance of a sun and granted with credits. To promote generalization of the learned strategies into daily life, transfer trials were used. Transfer trials were presented without immediate visual feedback and were included from session 11 (25 %) and session 21 (50 %) onwards. To further transfer learned behaviors, participants were instructed to retrieve their neurofeedback experiences by watching printed graphics of the training during school and homework. Compliance was verified by questioning the participants whether they used the transfer cards over the intervention period. Transfer cards were used by 84 % of the participants. See also Supplement Appendix 2 for more detailed information about the neurofeedback intervention. The mean number of training sessions of participants who completed the assessments at post intervention (*n* = 38) was 29 (*M* = 28.53,* SD* = 2.63, range between 19 and 30).

*Medication (MPH).* After the pre-intervention assessment, a 4-week double-blind randomized placebo-controlled titration procedure was used to determine the optimal individual dose of short-acting methylphenidate (MPH) [38]. The 4-week titration phase was preceded by a baseline week to determine ADHD symptoms without MPH, and was followed by a lead-in week in which on three consecutive days, twice-daily (at breakfast and lunch time), doses of (1) 5 mg, (2) 10 mg, and (3) 15 mg (<25 kg body weight) or 20 mg MPH (>25 kg body weight) were used to assess possible adverse effects. During the 4 weeks titration phase, children received in pseudo-random order (1) 5 mg, (2) 10 mg, (3) 15 mg or 20 mg MPH or (4) placebo for 1 week, twice daily. During the titration phase, children, parents and teacher as well as the researchers were blind with regard to the prescribed dose. At the end of each week, parents and teacher were asked to evaluate inattention and hyperactivity/impulsivity symptoms on the DBDRS, and adverse effects on the MTA Side Effects Rating Scale [39]. In total, 31 children completed the titration procedure. Children were classified by a standardized procedure [40] as responders when their ADHD symptoms significantly decreased compared to placebo (*n* = 29). The standardized procedure [40] classified children as non-responders when they did not show any decrease in inattention and hyperactivity/impulsivity symptoms across MPH doses and placebo as compared to baseline assessments (*n* = 2). When children were found to respond equally well across different MPH doses, the lowest MPH dose was prescribed. The two non-responders were treated with 5 mg MPH twice daily. The child’s psychiatrist prescribed the optimal dose for the remaining intervention period (5 mg to 10 children including 8 responders and 2 non-responders, 10 mg to 14 children, 15 mg to 2 children, and 20 mg to 5 children).

*Physical activity (PA) as semi-active control condition.* Maximum heart rate (HRmax) was determined before the start of the first training session. Each training session started with 5 min of warming up, followed by five 2-min moderate intensity exercises at a level of 70–80 % of HRmax. After a 5-min break, five 2-min vigorous intensity exercises of 80–100 % of HRmax were performed. Each training finished with a 5-min cool down. Time and heart rate were monitored and registered using a POLAR FT4 watch (Polar Electro Oy, Kempele, Finland). The mean number of sessions of participants who completed the assessments at post intervention (*n* = 34) was 28 (*M* = 27.74,* SD* = 3.56, range 12–30).

### Outcome measures

The *auditory oddball task* was used to measure attention [41]. This task contained 255 standard tones (523 Hz, 85 %) and 45 target tones (1046 Hz, 15 %), presented pseudo-randomly for 100 ms. Children were instructed to attend to the stimuli and to press a button on a response box with the right index finger when they heard a target. Outcome measures were response speed (mean reaction time; MRT), assessing attention, and the coefficient of variation (CV) [CV = MRT SD/MRT], a measure of attentional lapses [42]. Omission and commission errors were uncommon and, therefore, excluded from analyses.

The *stop*-*signal task (SST)* was primarily used to measure inhibition [43]. This task required children to perform a binary-choice reaction time task using visual stimuli (go stimuli). Children were instructed to inhibit their response when a go stimulus was followed by a visual stop signal. A full description of the task can be found in Janssen et al. [44]. Variables of interest were: (1) stop-signal reaction time (SSRT), a measure of the speed of the inhibitory process, calculated by subtracting mean stop-signal delay (SSD) from MRT; (2) number of commission on go trials, measuring impulsivity; (3) number of omission errors on go trials, assessing attention; (4) response speed (MRT), and (5) variability of response speed as calculated by coefficient of variation (CV), measuring lapses of attention.

The *visual spatial working memory task (VSWM)* [45, 46] was assessed to measure short-term storage or maintenance of visual-spatial information (forward condition) and visuospatial working memory (backward condition). Children were instructed to repeat sequences of yellow circles, presented on a computer screen in a 4 × 4 grid, in a forward order (forward condition) and a reversed order (backward condition). Variables of interest were the number of correct trials per condition.

### Procedure

The study was approved by the national medical ethics committee (NL 31641.029.10 CCMO). Written informed consent was obtained before participation from all parents and children aged 11 and older.

Pre-intervention (t0) assessment took place in the week prior to the start of the intervention. Post-intervention (t1) assessment took place 1 week after the last training session. Part of the data of this study are presented elsewhere [34, 47]. During t1 assessment, the MPH-group continued use of medication. Due to technical problems or misinterpretation of the task, data of 23 participants for the oddball task and 10 participants for the stop-signal task were not available for analysis. Finally, data of 89 participants for the oddball task (NFB *n* = 30; MPH *n* = 29; PA *n* = 30) and 102 participants for the stop signal task (NFB *n* = 36; MPH *n* = 33; PA *n* = 33) were analyzed. Interventions took place between September 2010 and March 2014.

### Statistical methods

Statistical analyses were performed with the IBM SPSS Statistics, version 20.0 [48]. Differences between treatment groups in terms of background characteristics were analyzed with a Chi-square test or ANOVA with Tukey post hoc tests. Group characteristics and outcome measures were subjected to attrition analyses using ANOVA, comparing the initially randomized sample to the sample that completed the interventions.

To compare treatment effects, General Linear Model (GLM) repeated measures (RM) ANOVAs were applied, with time [between pre-intervention (t0) and post-intervention (t1)] as within-subject factor and group (NFB, MPH and PA) as between-subject factor. For these analyses, the adjusted difference at post-intervention [ADt1-t0] and accompanying 95 % confidence interval (95 % CI) and the accompanying effect size (partial eta squared, *η*_p_^2^) are reported. Effect sizes are expressed in percentage of explained variance in partial eta squared (*η*_p_^2^; with thresholds for small, medium, and large effects corresponding to *η*_p_^2^ = 0.01, *η*_p_^2^ = 0.06, and *η*_p_^2^ = 0.14, respectively [49]. In case of significant time by group interactions, post hoc two-way between-groups interactions analyses were performed separately for the between-subject factors (1) NFB and MPH, (2) MPH and PA and (3) NFB and PA with time (t0, t1) as within-subject factor. Only significant results of *p* ≤ 0.05 are reported. Intention-to-treat analyses were performed using imputation with Last Observation Carried Forward (LOCF). Complete case analyses were performed for participants who completed pre- and post-intervention assessments. Post hoc analyses were performed, with separate addition of assessment site (Amsterdam or Rotterdam) and comorbid disorders (ADHD or ADHD with comorbid disorders). At group level, we found participants in the neurofeedback training were getting better at decreasing theta/beta ratio over time [50]. Therefore, to investigate the relation between getting better at decreasing theta/beta ratio (EEG slopes) and improvement in cognitive measures (difference scores), Pearson correlations were computed. Decreased theta/beta ratio was represented by (1) theta slopes over runs (within sessions) and theta slopes over sessions and (2) beta slopes over runs and theta slopes over sessions. Improvement in cognitive measures was defined by difference scores (t1-t0) of the variables of interest as described above.

## Results

### Group characteristics

At pre-intervention, group characteristics, and both behavioral and neurocognitive measures did not significantly differ between treatment groups (see Table 1).

**Table 1:** Group characteristics assessed pre-intervention (t0)

	Total	NFB	MPH	PA	GROUP		
					*df*	*F*	*p*
*n*	112	39	36	37			
Age in years, M (SD)	9.63 (1.76)	9.96 (1.88)	9.11(1.26)	9.80 (1.96)	(2,109)	2.48	0.09
Gender, M/F	85/27	30/9	27/9	28/9		0.04^a^	0.98
IQ, M (SD)	99.75 (13.36)	100.56 (13.18)	101.11 (14.24)	97.57 (12.74)	(2,109)	0.75	0.48
Parent ratings							
DBDRS							
Inattention (SD)	16.24 (5.30)	16.56 (5.10)	16.33 (5.65)	15.81 (5.26)	(2,109)	0.20	0.82
H/I (SD)	13.73 (6.12)	14.31 (6.03)	13.42 (6.40)	13.43 (6.03)	(2,109)	0.26	0.77
SDQ (SD)	16.60 (4.26)	16.90 (4.54)	15.64 (4.23)	17.22 (3.93)	(2,109)	1.41	0.25
SWAN							
Inattention (SD)	1.36 (0.64)	1.42 (0.52)	1.39 (0.70)	1.28 (0.70)	(2,109)	0.51	0.61
H/I (SD)	1.24 (0.75)	1.30 (0.70)	1.14 (0.72)	1.28 (0.82)	(2,109)	0.48	0.62
SDSC	45.45 (10.78)	45.32 (10.55)	45.09 (9.11)	44.97 (12.70)	(2,105)^b^	0.06	0.94
Teacher ratings							
DBDRS							
Inattention (SD)	16.25 (5.78)	15.56 (5.36)	17.61 (6.30)	15.65 (5.63)	(2,109)	1.48	0.23
H/I (SD)	13.33 (8.07)	14.13 (7.12)	12.75 (9.70)	13.05 (7.44)	(2,109)	0.30	0.74
SDQ (SD)	14.65 (5.14)	14.51 (4.71)	13.48 (5.43)	15.91 (5.17)	(2,104)^c^	1.96	0.15
SWAN							
Inattention (SD)	1.43 (0.75)	1.40 (0.90)	1.52 (0.62)	1.38 (0.69)	(2,104)^c^	0.34	0.71
H/I (SD)	1.08 (1.03)	1.18 (0.92)	0.93 (1.25)	1.12 (0.92)	(2,104)^c^	0.58	0.56

*y* years, *DBDRS* disruptive behaviour disorder rating scale, *H/I* hyperactivity/impulsivity scale, *IQ* intelligence quotient, *SDQ* strength and difficulty Questionnaire, *SWAN* strengths and weakness of ADHD symptoms and normal behaviour scale, *SDSC* sleep disturbance scale

^a^χ(2)

^b^NFB, *n* = 38; MPH, *n* = 35; PA, *n* = 35

^c^NFB: *n* = 39; MPH: *n* = 33; PA: *n* = 35

### Attrition analysis

No differences were found in group characteristics and pre-intervention measures between the participants as randomized and the participants who completed the intervention.

### Intention-to-treat analyses

Table 2 presents the results of the neurocognitive treatment effects for the three intervention groups and statistical results of the group analyses.

**Table 2:** Results of intention-to-treat analyses for the neurocognitive measures

	*n*	Pre-intervention (*t*0)	Post-intervention (*t*1)	Adjusted difference [95 % CI] at post-intervention *t*1–*t*0	Time (*t*0–*t*1)				Time by Group				Post Hoc multiple sites over time^a^				Post Hoc ADHD + ADHD and comorbid disorders over time^a^			
		M (SD)	M (SD)	*AD*	*df*	*F*	*p*	η_p_^2^	*df*	*F*	*p*	η_p_^2^	*df*	*F*	*p*	η_p_^2^	*df*	*F*	*p*	η_p_^2^
Oddball task																				
MRT					(1,86)	10.20	0.002	0.11	(2,86)	11.74	<0.001	0.21	(2,85)	11.68	<0.001	0.22	(2,85)	11.11	<0.001	0.21
NFB	30	440.72 (100.00)	433.54 (95.63)	−7.20 [−25.64, 11.30]																
MPH	29	461.56 (68.65)	404.40 (63.00)	−57.19 [−81.60, −32.80]																
PA	30	438.01 (88.51)	447.02 (90.06)	9.01 [−9.33, 27.35]																
CV				−0.02 [−0.03, −0.01]	(1,86)	5.73	0.019	0.06	(2,86)	0.22	0.803	0.01	(2,85)	0.17	0.841	<0.01	(2,85)	0.14	0.867	<0.01
NFB	30	0.29 (0.08)	0.28 (0.07)																	
MPH	29	0.28 (0.04)	0.26 (0.06)																	
PA	30	0.29 (0.07)	0.28 (0.07)																	
Stop-signal task																				
SSRT					(1,99)	29.35	<0.001	0.23	(2,99)	10.56	<0.001	0.18	(2,98)	10.00	<0.001	0.17	(2,98)	10.41	<0.001	0.18
NFB	36	271.39 (76.00)	252.89 (83.60)	−19.03 [−38.42, 0.36]																
MPH	33	278.10 (91.40)	202.30 (96.20)	−94.92 [−123.90, −65.94]																
PA	33	245.56 (84.83)	236.54 84.06)	−9.02 [−29.79, 11.74]																
Commission					(1,99)	5.80	0.018	0.06	(2,99)	5.05	0.008	0.09	(2,98)	5.29	0.007	0.10	(2,98)	5.29	0.007	0.10
NFB	36	20.22 (14.00)	18.20 (15.03)	−2.10 [−5.93, 1.82]																
MPH	33	19.70 (11.35)	12.64 (9.13)	−7.03 [−10.60, −3.50]																
PA	33	18.24 (10.14)	19.61 (12.00)	1.36 [−2.48, 5.20]																
Omission					(1,99)	3.90	0.051	0.04	(2,99)	7.62	0.001	0.13	(2,98)	7.05	0.001	0.13	(2,98)	7.52	0.001	0.13
NFB	36	16.00 (13.67)	13.67 (14.19)	−2.33 [−5.85, 1.19]																
MPH	33	14.18 (11.05)	7.12 (9.83)	−7.06 [−10.84, −3.30]																
PA	33	14.64 (12.25)	17.80 (16.15)	3.15 [−0.70, 7.00]																
MRT				−32.40 [−49.51, −15.28]	(1,99)	14.12	<0.001	0.13	(2,99)	1.43	0.245	0.03	(2,98)	1.56	0.216	0.03	(2,98)	1.38	0.257	0.03
NFB	36	642.68 (123.71)	610.12 (122.39)																	
MPH	33	679.78 (122.31)	629.35 (136.23)																	
PA	33	631.73 (110.42)	617.52 (123.86)																	
CV				−0.01 [−0.02, <−0.01]	(1,99)	4.64	0.034	0.05	(2,99)	0.59	0.556	0.01	(2,98)	0.54	0.587	0.01	(2,98)	0.58	0.562	0.01
NFB	36	0.28 (0.04)	0.27 (0.05)																	
MPH	33	0.27 (0.03)	0.26 (0.05)																	
PA	33	0.28 (0.04)	0.28 (0.04)																	
VSWM																				
Forward				0.71 [0.24, 1.17]	(1,109)	9.07	0.003	0.08	(2,109)	1.09	0.341	0.08	(2,108)	1.07	0.347	0.02	(2,108)	1.08	0.345	0.02
NFB	39	12.26 (2.92)	12.67 (3.60)																	
MPH	36	11.00 (2.58)	12.17 (2.72)																	
PA	37	11.16 (2.73)	11.68 (3.53)																	
Backward				1.32 [0.78, 1.86]	(1,109)	22.20	<0.001	0.17	(2,109)	1.32	0.272	0.02	(2,108)	1.30	0.276	0.02	(2,108)	1.33	0.270	0.02
NFB	39	10.90 (3.08)	11.67 (3.40)																	
MPH	36	9.58 (2.50)	11.33 (3.60)																	
PA	37	9.95 (2.95)	11.00 (3.32)																	

*MRT* mean reaction time, *CV* coefficient of variation, *SSRT* stop-signal reaction time, *VSWM* visual spatial working memory

^a^Post hoc analyses for multiple sites and comorbid disorders seperately

Results on the oddball task, used to measure attention, showed a group by time interaction for MRT. Post-hoc analyses revealed that the MPH group showed greater reductions of MRT over time than the NFB group, *F*(1,57) = 11.29, *p* = 0.001, *η*_p_^2^ = 0.17, and PA group, *F*(1,57) = 19.90, *p* < 0.001, *η*_p_^2^ = 0.26, suggesting enhanced attention in the MPH group compared to the NFB and PA group. NFB and PA did not differ from each other, *F*(1,58) = 1.62, *p* = 0.21, *η*_p_^2^ = 0.03. A main effect of time was found for CV in the absence of a significant group by time interaction, indicating that all groups improved equally over time on this measure of attentional lapses.

A group by time interaction was found for SSRT measured with the SST. Post-hoc analyses revealed a greater reduction in SSRT for the MPH group than for both the NFB group, *F*(1,67) = 12.73, *p* = 0.001, *η*_p_^2^ = 0.16, and the PA group, *F*(1,64) = 15.76, *p* < 0.001, *η*_p_^2^ = 0.20, indicating faster inhibitory control processes in children in the MPH group compared to both the NFB and PA group. No differences were found between the NFB and PA group on SSRT, *F*(1,67) = 0.47, *p* = 0.49, *η*_p_^2^ < 0.01. Results of other measures of interest obtained from the SST, revealed a group by time interaction for commission errors. Post-hoc analyses demonstrated a trend towards larger decrease in commission errors for the MPH group than the NFB group, *F*(1,67) = 3.67, *p* = 0.060, *η*_p_^2^ = 0.05. Furthermore, a significant larger decrease in commission errors was found for the MPH group compared to the PA group, *F*(1,64) = 10.72, *p* = 0.002, *η*_p_^2^ = 0.14, together suggesting decreased impulsivity in the MPH group. No differences were found between the NFB and PA group on commission errors, *F*(1,67) = 1.62, *p* = 0.21, *η*_p_^2^ = 0.02. A group by time interaction was also found for omission errors. Post-hoc analyses demonstrated a trend towards a larger decrease in omission errors for the MPH group than for the NFB group, *F*(1,67) = 3.47, *p* = 0.067, *η*_p_^2^ = 0.05. In addition, a larger decrease in omission errors was found for the MPH group compared to the PA group, *F*(1,64) = 14.85, *p* < 0.001, *η*_p_^2^ = 0.19, suggesting improved attention in the MPH group. Analyses comparing the NFB and PA group on omission errors revealed a different pattern per group, *F*(1,67) = 4.60, *p* = 0.036, *η*_p_^2^ = 0.06, with a slight (non-significant) increase in omission-errors over time for the PA group and a slight (non-significant) decrease in omission-errors over time for the NFB group. Main effects of time, without time by group interactions, were found for the other two variables of attention, MRT and CV. These results indicate equal improvements in all groups, with faster reaction times and less variable response speed at post-intervention compared to pre-intervention.

Results of the VSWM revealed a main effect of time on the forward and backward condition. In the absence of significant group by time interactions, indicating similar improvements over time for all three groups on short-term storage and working memory.

### Complete case analyses

All analyses were rerun using complete case analysis and all significant findings were replicated with two exceptions: complete case analyses showed a significant larger decrease in both commission errors, *F*(1,61) = 5.63, *p* = 0.021, *η*_p_^2^ = 0.08, and omission errors, *F*(1,61) = 5.36, *p* = 0.024, *η*_p_^2^ = 0.08, for the MPH group compared to the NFB group, whereas these differences just escaped conventional levels of significance in the intention-to-treat analyses. See also the Supplement Appendix 3 Table 1. Results of complete case analyses for neurocognitive measures.

### Post hoc analyses for assessment location and comorbid disorders

Children who were assessed and had their intervention in Amsterdam did not differ over time from children who were assessed and had their intervention in Rotterdam on the neurocognitive outcome measures. Similarly, children with ADHD did not differ over time from children with ADHD and comorbid disorders.

### Post hoc analyses EEG slopes and neurocognitive change

Analyses showed one negative correlation between beta slopes over sessions and change in SSRT (inhibitory control) as measured with the SST, *r*(36) = −0.39, *p* = 0.02. This result demonstrates that increase of beta over sessions correlates with improved inhibitory control at post-intervention. See also the Supplement Appendix 4 Table 2.

## Discussion

The present study compared neurofeedback as a stand-alone treatment to both stimulant medication and physical activity, acting as a semi-active control condition, on attention, inhibition and working memory. These neurocognitive functions are often impaired in children with ADHD [10] and play key roles in explanatory models of the disorder [51]. Results of the current study indicated superior effects of stimulant medication compared to both neurofeedback and physical activity on our measure of attention, as shown by faster response speed on the oddball task in children taking methylphenidate than in children who received neurofeedback or physical activity training. Compared to both neurofeedback and physical activity, stimulant medication also had superior effects on inhibitory control, measured by stop-signal reaction time of the stop signal task. Working memory, as measured by visual spatial working memory, showed similar improvements from pre- to post-intervention across all three groups. Overall, the effects of neurofeedback on neurocognitive functioning were comparable to the effects of our physical activity training acting as semi-active control condition.

One of the core deficits observed in ADHD are attention problems. In the current study, attentional functioning, as measured by response speed during the oddball task, showed greater improvements in children with ADHD receiving stimulant medication than in those receiving neurofeedback or physical activity training. This result is in line with the behavioral findings of the current study, showing superior effects of stimulant medication compared to neurofeedback in reducing parent as well as teacher reported attention problems [34]. Our findings are in line with the results of the only other available study comparing neurofeedback and stimulant medication on a task measuring attentional functioning [31]. The study of Ogrim and Hestad [31] showed greater improvements in omission errors and reaction time variation on the Visual Continuous Performance Task with stimulant medication than with neurofeedback. However, the three groups in the current study did not differ on the coefficient of variation in the oddball task. Indicating that although stimulant medication induced faster reaction times, the reactions did not become less variable. Although this seems in contradiction to the study of Ogrim and Hestad [31], note that the reaction time variation in the study of Ogrim and Hestad [31] did not control for the influence of response speed, whereas the current study did control for the influence of response speed using the coefficient of variation. Furthermore, our findings are in line with the results of two double blinded RCTs testing the effects of neurofeedback on a variety of attention paradigms, failing to demonstrate benefits of neurofeedback on attentional functioning compared to sham-neurofeedback in children with ADHD [19, 23]. Only the study by Bakhshayesh and colleagues [20] revealed superior effects of neurofeedback compared to EMG-biofeedback on neurocognitive measures of attention in children with ADHD using a single-blinded RCT design. To conclude, with the exception of the study by Bakhshayes et al. [20], all available studies support the conclusion of the current study, indicating that the effects of neurofeedback are insufficient to bring about improved attention as measured with neurocognitive tasks.

Similar to our findings on attention, stimulant medication induced larger improvements in inhibition, as reflected by decreased stop-signal reaction times (SSRT), compared to both the neurofeedback and our semi-active control condition. This result is in accordance with our earlier EEG power spectra [47] and event-related potential (ERP) findings [52], indicating specific neurophysiological effects during the stop task for stimulant medication compared to neurofeedback and physical activity while no effects were found for neurofeedback compared to physical activity. More specifically, ERP results indicated that medication induced increase in P3 amplitude strongly correlated with improved SSRT (*r* = −0.625). Inhibitory control deficits have been considered as one of the central deficits in ADHD [3]. Our results of improved inhibition with stimulant medication as compared to neurofeedback, are in line with behavioral results presented in the review of Arns et al. [53]. That review evaluated the effects of neurofeedback in ADHD and concluded that the effect size for neurofeedback on symptoms of impulsivity was significantly lower compared to the effect size for methylphenidate on impulsivity [53]. In addition, the current study found comparable effects in inhibition in neurofeedback and the semi-active control condition, suggesting that neurofeedback does not impact on inhibition. Taken together, we conclude that neurofeedback has no specific effects on inhibition.

Both short-term storage and visuospatial working memory improved from pre- to post-intervention across all three groups. In line with our findings, studies comparing neurofeedback to sham-neurofeedback found comparable effects on verbal working memory over time [23, 28]. Furthermore, the study of Bink et al. [21], comparing treatment as usual combined with neurofeedback to treatment as usual, found similar improvements on verbal working memory. Our findings, combined with results of these previous studies [21, 23, 28], suggest that the improvement of working memory in children is related to practice effects which occur due to multiple testing, a problem that has often been reported with repeated testing [54]. Note that apart from these practice effects found for working memory, we did find superior effects of stimulant medication on measures of attention, inhibition, and impulsivity, indicating that the instruments used in the current study proved sensitive to treatment effects.

Overall, the present study found larger improvements in neurocognitive functioning with stimulant medication than with neurofeedback. The present study applied a double blind titration procedure to determine an optimal dose of stimulant medication while the neurofeedback intervention was not optimized to the child’s individual needs. Therefore, one could argue that the results we found were due to the supremacy of the titration protocol. However, compared to the semi-active control condition we found no superiority of neurofeedback. On top of that, there is an ongoing debate on the efficacy of various neurofeedback protocols. The current study used the theta/beta training protocol. This protocol is based on findings of increased theta (4–8 Hz) and decreased beta (13–20 Hz) in ADHD [16] and is often used in ADHD treatment with the aim to improve attention [18]. The question, however, is whether this protocol is effective for the treatment of neurocognitive dysfuntioning in children with ADHD. Bink et al. [21] already pointed out that the majority of studies that failed to show any effects of neurofeedback, used a somewhat different protocol compared to the study of Bakhshayes et al. [20], the only single-blinded study that found improvements on neurocognitive attention measures thus far. In the study of Bakhshayes et al. [20], a neurofeedback protocol was used which rewarded not only suppression of theta, but also high beta (16–20 Hz). Rewarding these higher beta-band frequencies may underlie the positive effects found in the study of Bakhshayes et al. [20]. However, despite the fact that the neurofeedback training used in the current study also encompassed higher beta frequencies (16–20 Hz), we found no positive effects for neurofeedback compared to stimulant medication.

The present study examined the effects of neurofeedback on neurocognitive functioning compared to stimulant medication as well as a semi-active control condition. As we found theta and beta learning effects in participants that received neurofeedback [50], we were interested in exploring the relation between these learning effects and improvement in cognitive measures. Results showed one significant relation between increase of beta over sessions and improved inhibitory control at post intervention. However, note that we might have a multiple testing problem as we tested 36 correlations and found only one significant outcome, which may be a chance finding. Therefore, this result should be interpreted with caution. Further, this RCT study successfully allocated participants randomly to the three intervention groups and sample sizes were adequate to detect medium sized effects. Still, a few points need consideration when interpreting the current findings. First, in the current study, physical activity was implemented as a semi-active control condition where frequency and intensity of the training were similar to the neurofeedback intervention. The review of Halperin, Berwid, and O’Neill [55] suggested positive effects with more intensive physical activity on children’s ADHD-related behaviors. Thus, it might be argued that our semi-active control condition might have exerted beneficial effects on neurocognitive functioning of our participants, and thus might not have been the optimal comparison condition. However, children in the physical activity intervention received only 2-min bounds of physical activity during a time period of only 20 min. The use of 2-min bounds of physical activity does not correspond with the recommendations on physical activity found in the literature [55]. Therefore, it does not seem likely that the physical activity protocol we used exerted beneficial effects on neurocognitive functioning. More research on physical activity is necessary to substantiate its possible chronic effects on the problem behavior of children with ADHD. Second, it could be argued that expectation might have had a large influence on the results [56]. Therefore, expectations of parents and teachers at pre-intervention were assessed. Results showed that only in the neurofeedback intervention, parents with higher treatment expectations of neurofeedback rated their child as more improved in terms of inattentive symptoms. Stimulant medication and physical activity revealed no association between expectancy and reported changes [34]. Third, the current study found superior effects of stimulant medication on neurocognitive functioning compared to a neurofeedback training of theta/beta frequencies in children with ADHD. However, the proposition that increased theta/beta ratio may be considered as biomarker for ADHD has been challenged by recent research [57, 58] and other neurofeedback protocols such as slow cortical potentials (SCP) training are suggested for the treatment of ADHD [59]. SCP is not the only alternative neurofeedback protocol. Because frequency band and SCP neurofeedback are often criticized for the large time investment, with many studies using 30 sessions, new neurofeedback methods such as near-infrared spectroscopy (NIRS) are proposed. A recent pilot study compared the effects of 12 sessions of SCP and NIRS, and found promising effects for NIRS on parent-, teacher ratings and improvement on an attention test [60]. Nevertheless, the effectiveness of both SCP and NIRS as a treatment for children with ADHD needs to be confirmed by randomized controlled studies using larger sample sizes. Clearly, more research on the specificity of the various neurofeedback protocols is recommended.

In conclusion, stimulant medication showed superior effects over neurofeedback on improving neurocognitive functions, and in particular on attention and inhibition. The effects of neurofeedback on neurocognitive functioning were comparable to the semi-active control condition, indicating that for neurofeedback showed no specific effects on neurocognitive functioning. Hence, results of the current RCT do not support the use of theta/beta training as a stand-alone treatment to improve neurocognitive functioning in children with ADHD.

## Electronic supplementary material

Below is the link to the electronic supplementary material.

Supplementary material 1 (DOC 220 kb)

Supplementary material 2 (DOC 28 kb)

Supplementary material 3 (DOC 127 kb)

Supplementary material 4 (DOC 64 kb)
