# Supplementary material for: An RCT into the effects of neurofeedback on neurocognitive functioning compared to stimulant medication and physical activity in children with ADHD
Source: Eur Child Adolesc Psychiatry. 2016 Sep 24;26(4):457–68. doi: 10.1007/s00787-016-0902-x (PMC5364239; doi:10.1007/s00787-016-0902-x)
Supplement: Supplementary file 2 — Supplementary material 2 (DOC 28 kb) [file 787_2016_902_MOESM2_ESM.doc]

Supplement Appendix 2

**Methods**

*Interventions*

*Neurofeedback*. The THERAPRAX**®** EEG Biofeedback system (Neuroconn GmbH, Germany) with a DC-amplifier and a sampling rate of 128Hz was used to transmit and analyze the EEG signal. Reference and ground electrodes were attached to right and left mastoids, respectively. Electro-oculogram was obtained with two electrodes at external canthi, and two electrodes at supra- and infraorbital sides. Ocular correction was applied as described in Schlegelmilch et al.(2004). Subsequently, a theta/beta index [theta(μV/Hz)-beta(μV/Hz)/theta(μV/Hz)+beta(μV/Hz) was computed with a short-time-fourier transformed moving average for direct feedback.

Each training session started with a 1-minute baseline theta/beta index measurement, followed by 10 runs of neurofeedback.. Each run comprised four 30-second epochs. The first run of the first training started on a training level with the aim to reduce the theta/beta index with 3%. The training level increased or decreased based on performance of former runs and could range between 3-52%, relative to training session baseline, over the total treatment period of 10 weeks. Number of credits per trial depended on the training level, with more credits for higher levels.
