# Supplementary material for: An RCT into the effects of neurofeedback on neurocognitive functioning compared to stimulant medication and physical activity in children with ADHD
Source: Eur Child Adolesc Psychiatry. 2016 Sep 24;26(4):457–68. doi: 10.1007/s00787-016-0902-x (PMC5364239; doi:10.1007/s00787-016-0902-x)
Supplement: Supplementary file 3 — Supplementary material 3 (DOC 127 kb) [file 787_2016_902_MOESM3_ESM.doc]

Note. ^a^MRT=mean reaction time; ^b^CV=coefficient of variation; ^c^SSRT=stop-signal reaction time; ^d^VSWM=visual spatial working memory;

| Supplement Appendix 3 Table 1. Results of complete case analyses for the neurocognitive measures | | | | | | | | | | | | |  | |
| --- | --- | --- | --- | --- | --- | --- | --- | --- | --- | --- | --- | --- | --- | --- |
|  |  |  | Pre-  Intervention (t0) | Post-Intervention (t1) | Adjusted difference  [95% CI] at post-intervention t1-t0 | Time (t0 to t1) | | | | Time x Group | | | | |
|  |  | n | M(SD) | M(SD) | *AD* | df | F | *p* | η_p_^2^ | df | F | *p* | | η_p_^2^ |
| **Oddball task** |  |  |  |  |  |  |  |  |  |  |  |  | |  |
| MRT^a^ |  |  |  |  |  | (1,77) | 12.75 | 0.001 | 0.14 | (2,77) | 14.15 | <0.001 | | 0.27 |
|  | NFB | 29 | 443.81(100.24) | 436.39(96.02) | -7.42[-26.55, 11.70] |  |  |  |  |  |  |  | |  |
|  | MPH | 24 | 462.15(69.15) | 393.06(55.69) | -69.09[-96.29, -41.88] |  |  |  |  |  |  |  | |  |
|  | PA | 27 | 432.74(89.00) | 442.75(91.26) | 10.01[-10.47, 30.49] |  |  |  |  |  |  |  | |  |
|  |  |  |  |  |  |  |  |  |  |  |  |  | |  |
| CV^b^ |  |  |  |  | -0.02[-0.04, <-0.01] | (1,77) | 6.00 | 0.017 | 0.07 | (2,77) | 0.34 | 0.714 | | 0.01 |
|  | NFB | 29 | 0.29(0.09) | 0.28(0.07) |  |  |  |  |  |  |  |  | |  |
|  | MPH | 24 | 0.28(0.07) | 0.25(0.07) |  |  |  |  |  |  |  |  | |  |
|  | PA | 27 | 0.29(0.07) | 0.28(0.07) |  |  |  |  |  |  |  |  | |  |
| **Stop-signal task** |  |  |  |  |  |  |  |  |  |  |  |  | |  |
| SSRT^c^ |  |  |  |  |  | (1,90) | 38.38 | <0.001 | 0.30 | (2,90) | 15.49 | <0.001 | | 0.26 |
|  | NFB | 35 | 273.39(76.12) | 254.36(84.34) | -19.03[-38.42, 0.36] |  |  |  |  |  |  |  | |  |
|  | MPH | 28 | 277.24(84.46) | 182.32(75.41) | -94.92[-123.90, -65.94] |  |  |  |  |  |  |  | |  |
|  | PA | 30 | 247.73(88.00) | 237.81(87.39) | -9.92[-32.87, 13.02] |  |  |  |  |  |  |  | |  |
| Comission |  |  |  |  |  | (1,90) | 7.24 | 0.009 | 0.07 | (2,90) | 6.28 | 0.003 | | 0.12 |
|  | NFB | 35 | 20.63(14.00) | 18.51(15.11) | -2.11[-6.10, 1.88] |  |  |  |  |  |  |  | |  |
|  | MPH | 28 | 20.68(11.47) | 11.93(9.43) | -8.75[-12.73, -4.77] |  |  |  |  |  |  |  | |  |
|  | PA | 30 | 18.00(10.40) | 19.47(12.40) | 1.50[-2.75, 5.75] |  |  |  |  |  |  |  | |  |
| Omission |  |  |  |  |  | (1,90) | 5.00 | 0.028 | 0.05 | (2,90) | 8.95 | <0.001 | | 0.17 |
|  | NFB | 35 | 16.37(13.69) | 13.97(14.28) | -2.40[-6.02, 1.22] |  |  |  |  |  |  |  | |  |
|  | MPH | 28 | 14.14(10.30) | 5.43(7.34) | -8.71[-13.00, -4.44] |  |  |  |  |  |  |  | |  |
|  | PA | 30 | 12.57(9.70) | 16.03(15.16) | 3.47[-0.78, 7.71] |  |  |  |  |  |  |  | |  |
|  |  |  |  |  |  |  |  |  |  |  |  |  | |  |
| MRT^a^ |  |  |  |  | -37.02[-55.65, -18.40] | (1,90) | 15.60 | <0.001 | 0.15 | (2,90) | 1.95 | 0.15 | | 0.04 |
|  | NFB | 35 | 647.40(122.18) | 613.91(122.00) |  |  |  |  |  |  |  |  | |  |
|  | MPH | 28 | 687.22(116.36) | 625.26(132.21) |  |  |  |  |  | |
|  | PA | 30 | 625.78(107.92) | 610.16(122.10) |  |  |  |  |  | |
| CV^b^ |  |  |  |  | -0.01[-0.02, <-0.01] | (1,90) | 5.02 | 0.027 | 0.05 | (2,90) | 0.72 | 0.49 | | 0.02 |
|  | NFB | 35 | 0.28(0.04) | 0.27(0.05) |  |  |  |  |  |  |  |  | |  |
|  | MPH | 28 | 0.27(0.03) | 0.25(0.05) |  |  |  |  |  |  |  |  | |  |
|  | PA | 30 | 0.28(0.03) | 0.27(0.03) |  |  |  |  |  |  |  |  | |  |
| **VSWM^d^** |  |  |  |  |  |  |  |  |  |  |  |  | |  |
| Forward |  |  |  |  | 0.79[0.29, 1.29] | (1,100) | 9.63 | 0.002 | 0.09 | (2,100) | 1.35 | 0.26 | | 0.03 |
|  | NFB | 38 | 12.32(2.93) | 12.74(3.58) |  |  |  |  |  |  |  |  | |  |
|  | MPH | 31 | 11.00(2.65) | 12.39(2.75) |  |  |  |  |  | |
|  | PA | 34 | 10.97(2.72) | 11.53(3.61) |  |  |  |  |  | |
|  |  |  |  |  |  |  |  |  |  | |
| Backward |  |  |  |  | 1.32[0.78, 1.86] | (1,100) | 23.63 | <0.001 | 0.19 | (2,100) | 1.80 | 0.17 | | 0.04 |
|  | NFB | 38 | 10.89(3.12) | 11.68(3.44) |  |  |  |  |  |  |  |  | |  |
|  | MPH | 31 | 9.68(2.54) | 11.71(3.66) |  |  |  |  |  | |
|  | PA | 34 | 9.62(2.81) | 10.76(3.32) |  |  |  |  |  | |
|  |  |  |  |  |  |  |  |  |  |  |  |  | |  |

Table 1. Group characteristics assessed pre-intervention (t0)

|  | | TOTAL | NFB | MPH | PA | GROUP | | |
| --- | --- | --- | --- | --- | --- | --- | --- | --- |
|  | |  |  |  |  | df | F | *p* |
| n | | 103 | 38 | 31 | 34 |  |  |  |
| Age in years, M (SD) | | 9.53 (1.70) | 9.87 (1.81) | 9.04 (1.24) | 9.60 (1.87) | (2,100) | 2.13 | .124 |
| Gender, M/F | | 78/25 | 29/9 | 24/7 | 25/9 |  | 0.15^a^ | .930 |
| IQ,^d^ M (SD) | | 99.99 (13.54) | 100.45 (13.34) | 101.6 (14.71) | 98.00 (12.80) | (2,100) | 0.61 | .547 |
| **Parent ratings** | |  |  |  |  |  |  |  |
| **DBDRS**^b^ | |  |  |  |  |  |  |  |
| Inattention (SD) | | 16.47(5.20) | 16.63(5.15) | 16.52(5.69) | 16.24(4.92) | (2,100) | 0.05 | .948 |
| H/I^c^ (SD) | | 13.63(6.00) | 14.50(5.99) | 13.16(6.00) | 13.09(6.05) | (2,100) | 0.63 | .535 |
| **SDQ**^e^ (SD) | | 16.79(4.14) | 16.76(4.52) | 16.03(4.15) | 17.50(3.69) | (2,100) | 1.02 | .366 |
| **SWAN**^f^ | |  |  |  |  |  |  |  |
| Inattention (SD) | | 1.39(0.63) | 1.44(0.51) | 1.39(0.73) | 1.33(0.68) | (2,100) | 0.27 | .761 |
| H/I (SD) | | 1.21(0.73) | 1.30(0.71) | 1.10(0.67) | 1.20(0.82) | (2,100) | 0.66 | .522 |
| **SDSC**^g^ | | 45.52(10.95) | 44.95(10.44) | 45.40(9.06) | 46.28(13.22) | (2,96)^h^ | 0.13 | .880 |
| **Teacher** | |  |  |  |  |  |  |  |
| **DBDRS**^b^ | |  |  |  |  |  |  |  |
| Inattention (SD) | | 16.15(5.84) | 15.37(5.29) | 17.48(6.55) | 15.79(5.71) | (2,100) | 1.22 | .301 |
| H/I^c^ (SD) | | 13.00(8.06) | 13.79(6.90) | 12.65(10.02) | 12.41(7.42) | (2,100) | 0.30 | .743 |
| **SDQ**^e^ (SD) | | 14.72(5.09) | 14.87(5.15) | 13.13(4.58) | 16.00(5.24) | (2,98)^i^ | 2.60 | .080 |
| **SWAN**^f^ | |  |  |  |  |  |  |  |
| Inattention (SD) | | 1.43(0.76) | 1.39(0.91) | 1.53(0.60) | 1.39(0.71) | (2,98)^i^ | 0.35 | .710 |
| H/I^c^ (SD) | | 1.07(1.04) | 1.15(0.91) | 0.94(1.30) | 1.09(0.93) | (2,98)^i^ | 0.37 | .694 |
|  | |  |  |  |  |  |  |  |
|  |  | | | | | | | |

Note. ^b^DBDRS=disruptive behaviour disorder rating scale; ^c^H/I=hyperactivity/impulsivity scale; ^d^IQ=intelligence quotient; ^h^NFB: *n*=37; MPH: *n*=30; PA: *n*=32; ^i^ NFB: *n*=38; MPH: *n*=30; PA: *n*=33; **^e^**SDQ=strength and difficulty questionnaire; **^g^**SDSC=sleep disturbance scale; **^f^**SWAN=strengths and weakness of ADHD symptoms and normal behaviour scale; ^a^χ(2); y=years.
