# Supplementary material for: An RCT into the effects of neurofeedback on neurocognitive functioning compared to stimulant medication and physical activity in children with ADHD
Source: Eur Child Adolesc Psychiatry. 2016 Sep 24;26(4):457–68. doi: 10.1007/s00787-016-0902-x (PMC5364239; doi:10.1007/s00787-016-0902-x)
Supplement: Supplementary file 4 — Supplementary material 4 (DOC 64 kb) [file 787_2016_902_MOESM4_ESM.doc]

Supplement Appendix 4 Table 2. Correlation between EEG slopes and neurocognitive change

|  | | theta slopes over runs | theta slopes over sessions | beta slopes over runs | beta slopes over sessions |
| --- | --- | --- | --- | --- | --- |
| OddBall task MRT^a^ | r | .083 | -.062 | .030 | -.167 |
|  | *p* | .668 | .748 | .877 | .386 |
|  | N | 29 | 29 | 29 | 29 |
| OddBall task CV^b^ | r | .152 | .074 | .168 | .043 |
|  | *p* | .431 | .704 | .384 | .823 |
|  | N | 29 | 29 | 29 | 29 |
| Stop-signal task SSRT^c^ | r | .315 | .044 | .000 | -.387 |
|  | *p* | .061 | .797 | 1.000 | .020 |
|  | N | 36 | 36 | 36 | 36 |
| Stop-signal task Comission | r | -.133 | .099 | -.040 | -.190 |
|  | *p* | .440 | .567 | .817 | .266 |
|  | N | 36 | 36 | 36 | 36 |
| Stop-signal task Omission | r | .087 | .014 | .135 | -.049 |
|  | *p* | .614 | .937 | .433 | .777 |
|  | N | 36 | 36 | 36 | 36 |
| Stop-signal task MRT^a^ | r | -.119 | -.224 | -.003 | .264 |
|  | *p* | .489 | .189 | .986 | .120 |
|  | N | 36 | 36 | 36 | 36 |
| Stop-signal task CV^b^ | r | -.024 | .317 | -.107 | -.088 |
|  | *p* | .891 | .060 | .535 | .609 |
|  | N | 36 | 36 | 36 | 36 |
| VSWM^d^ Forward | r | .074 | -.041 | -.007 | .026 |
|  | *p* | .660 | .809 | .965 | .876 |
|  | N | 38 | 38 | 38 | 38 |
| VSWM^d^ Backward | r | .093 | .055 | -.165 | -.107 |
|  | *p* | .579 | .743 | .323 | .523 |
|  | N | 38 | 38 | 38 | 38 |

Note. ^a^MRT=mean reaction time; ^b^CV=coefficient of variation; ^c^SSRT=stop-signal reaction time;

^d^VSWM=visual spatial working memory.
